# A Polymorphic 3’UTR Element in *ATP1B1* Regulates Alternative Polyadenylation and Is Associated with Blood Pressure

**DOI:** 10.1371/journal.pone.0076290

**Published:** 2013-10-01

**Authors:** Megana K. Prasad, Kavita Bhalla, Zhen Hua Pan, Jeffrey R. O’Connell, Alan B. Weder, Aravinda Chakravarti, Bin Tian, Yen-Pei C. Chang

**Affiliations:** 1 Division of Endocrinology, Diabetes and Nutrition, University of Maryland School of Medicine, Baltimore, Maryland, United States of America; 2 Marlene and Stewart Greenebaum Cancer Center, University of Maryland School of Medicine, Baltimore, Maryland, United States of America; 3 Department of Biochemistry & Molecular Biology, University of Medicine and Dentistry of New Jersey, New Jersey Medical School, Newark, New Jersey, United States of America; 4 Department of Internal Medicine, University of Michigan School of Medicine, Ann Arbor, Michigan, United States of America; 5 McKusick-Nathans Institute of Genetic Medicine, Johns Hopkins University School of Medicine, Baltimore, Maryland, United States of America; Institute of Molecular Medicine, Taiwan

## Abstract

Although variants in many genes have previously been shown to be associated with blood pressure (BP) levels, the molecular mechanism underlying these associations are mostly unknown. We identified a multi-allelic T-rich sequence (TRS) in the 3’UTR of *ATP1B1* that varies in length and sequence composition (T_22-27_ and T_12_GT _3_GT_6_). The 3’UTR of *ATP1B1* contains 2 functional polyadenylation signals and the TRS is downstream of the proximal polyadenylation site (A2). Therefore, we hypothesized that alleles of this TRS might influence *ATP1B1* expression by regulating alternative polyadenylation. *In vitro*, the T_12_GT _3_GT_6_ allele increases polyadenylation at the A2 polyadenylation site as compared to the T_23_ allele. Consistent with our hypothesis, the relative abundance of the A2-polyadenylated *ATP1B1* mRNA was higher in human kidneys with at least one copy of the T_12_GT _3_GT_6_ allele than in those lacking this allele. The T_12_GT _3_GT_6_ allele is also associated with higher systolic BP (beta = 3.3 mmHg, *p* = 0.014) and diastolic BP (beta = 2.4 mmHg, *p* = 0.003) in a European-American population. Therefore, we have identified a novel multi-allelic TRS in the 3’UTR of *ATP1B1* that is associated with higher BP and may mediate its effect by regulating the polyadenylation of the *ATP1B1* mRNA.

## Introduction

Hypertension (HTN) is a major public health concern affecting over 1 billion people worldwide, with hypertensive patients having an elevated risk of developing kidney disease, coronary disease, and stroke [1]. Genetic factors are involved in the development of HTN and heritability estimates for systolic and diastolic blood pressure (BP) measures range from 32%–68% [2,3,4,5]. Genome-wide linkage and association studies have uncovered novel loci involved in BP regulation and HTN pathophysiology [6,7,8]. However, the mechanisms by which genetic variants at these loci contribute to HTN are often poorly understood.

Previously, we identified a BP-related linkage region in 1q23-q32 and several single nucleotide polymorphisms (SNPs) associated with BP [9]. In humans, markers in 1q23–1q32 have been reproducibly linked with BP-related traits [10,11,12]. Homologous regions in mice and rats have also been shown to harbour BP-quantitative trait loci (QTLs) [13,14,15]. Of the biological candidate genes within this linkage region, we showed a 3’UTR SNP in *ATP1B1* (rs12079745) to be strongly associated with BP in a Caucasian population [9]. *ATP1B1* encodes the beta 1-subunit of Na-K ATPase, a ubiquitously expressed cotransporter essential for regulating cell volume, electrolyte and nutrient transport, and signal transduction [15]. In the kidney, Na-K ATPase provides the primary drive for Na and water reabsorption, which is necessary for the maintenance of fluid and electrolyte homeostasis [16]. Na-K ATPase consists of the catalytic alpha subunit, and the beta subunit, which is thought to be the “regulatory subunit” as it is the rate-limiting factor in the dimerization of Na-K ATPase, and is necessary for enzyme activity [17]. Indeed, SNPs in *ATP1B1* have also been found to be associated with BP in an African-American and in a Chinese population [18,19].

**Figure 1:**
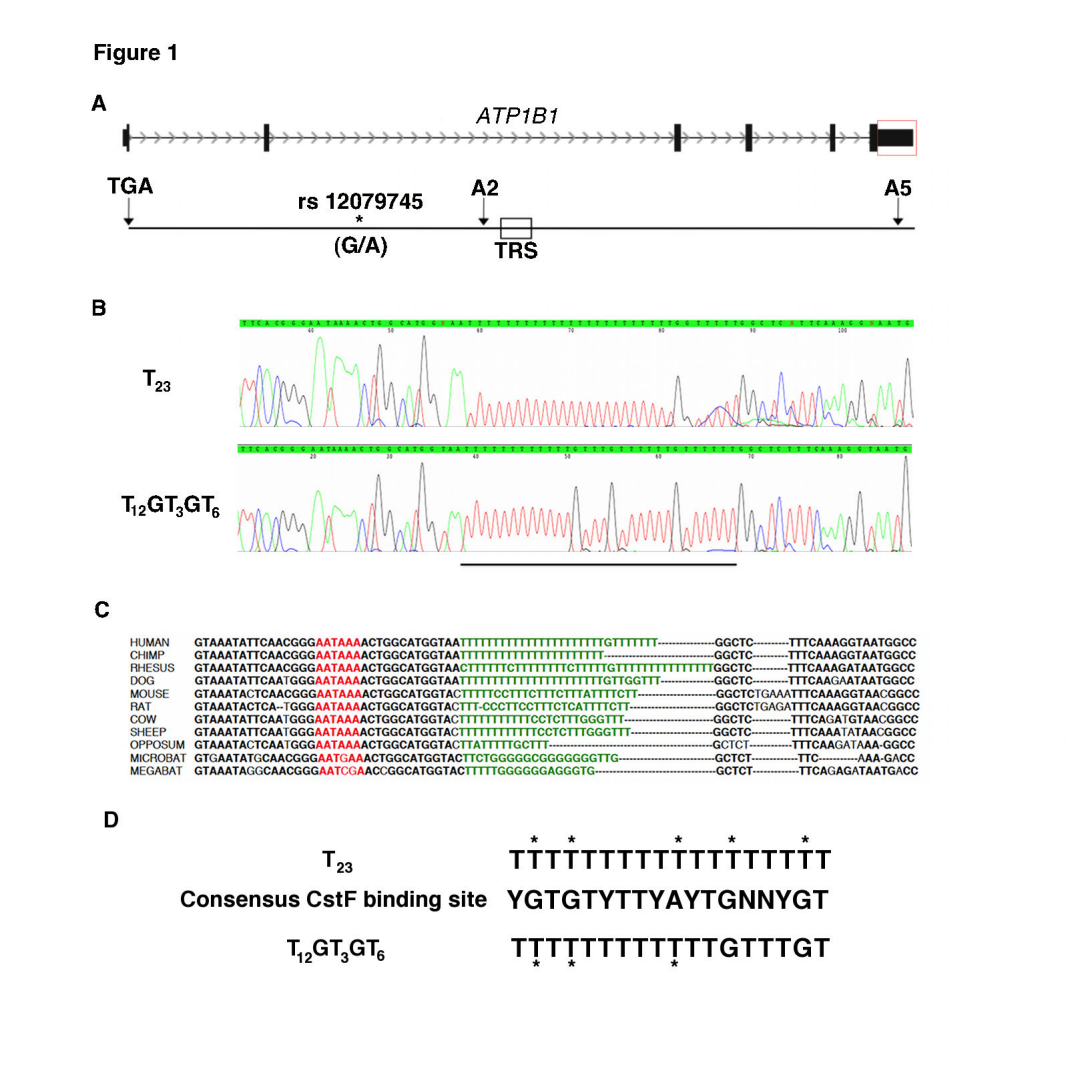
Structure and genotyping of the T-rich sequence in *ATP1B1*. A) Genomic architecture of the *ATP1B1* gene. The red box highlights the 3’UTR, which is enlarged and shown below. TGA: translational stop codon, *: G/A: SNP rs12079745, A2: A2 polyadenylation signal, TRS: T-rich element, A5: A5 polyadenylation signal. B) Representative Sanger sequencing chromatograms of the T_23_ and T _12_GT _3_GT_6_ alleles. Alleles are named based on the portion of the TRS that is unique to each allele (denoted by a horizontal bar). C) Sequence alignment of the TRS across mammals. The A2 polyadenylation signal and the TRS are highlighted in red and green respectively. D) Aligned are the 2a consensus sequence of the CstF binding site [38] and the sequences of the TRS with the T_23_ and T_12_GT _3_GT_6_ alleles. Asterisks mark the nucleotides in the TRS that deviate from the consensus CstF binding sequence. Y = C/T, N = A/C/G/T.

The human *ATP1B1* gene has 6 exons and encodes multiple mRNAs that differ in their 5’ and 3’UTR lengths due to the use of multiple transcription initiation and polyadenylation sites [20,21]. The structure of the 3’UTR of *Atp1b1* has been best studied in rats, in which the 3’UTR has 5 canonical polyadenylation sites, A1-A5, of which A1, A2, and A5 are predominantly used *in vivo* [21]. The differentially polyadenylated mRNAs are functionally distinct. The shorter A2-polyadenylated mRNA is translationally more efficient than the longer A5-polyadenylated mRNA due to a translational repressor sequence in the region unique to the A5-polyadenylated mRNA [22,23]. By contrast, the human *ATP1B1* gene has 4 canonical polyadenylation sites (rat A1 is absent) and only the A2 and A5 sites are used for the polyadenylation of the *ATP1B1* transcript (Figure 1A) [24]. Because the BP-associated SNP, rs12079745, is located in the 3’UTR near the A2 polyadenylation site, we hypothesized that sequence variation in the 3’UTR may regulate alternative polyadenylation, with alleles that promote the use of the A2 polyadenylation site leading to a translationally more efficient mRNA and an overall increase in *ATP1B1* expression.

Alternative polyadenylation plays an important role in regulating gene expression and thus contributes to normal development and disease [25]. Polyadenylation begins with the recognition of the canonical polyadenylation signal (either AAUAAA or AUUAAA in humans) and the less well-defined U/GU-rich core downstream element (DSE) by the cleavage and polyadenylation specificity factor (CPSF) and the cleavage stimulating factor (CstF), respectively [26]. It is believed that, upon forming a complex, CPSF and CstF recruit additional factors, including cleavage factors I and II and polyadenylate [poly(A)] polymerase, that together cleave the nascent pre-mRNA at the cleavage/polyadenylation site, between the polyadenylation signal and the DSE and add a poly(A) tail. Over 50% of human genes have multiple polyadenylation sites. In these genes, which site is used in a given mRNA is influenced by the surrounding *cis* elements, including the upstream sequence element, and the auxiliary DSE [26]. In addition, the abundance of cleavage and polyadenylation factors can also play a role in polyadenylation site choice [26]. While some non-canonical polyadenylation signals maintain normal function, other variants in these sequences alter their function and are the molecular bases of diseases. In contrast, because the DSE is less well characterized, very few disease-causing variants have thus been identified in the DSE [27].

Here, we report the identification of a novel polymorphic T-rich sequence (TRS) in the 3’UTR of *ATP1B1*, downstream of the A2 polyadenylation signal. Based on sequence composition and distance to the A2 polyadenylation signal, this TRS (U-rich sequence in the corresponding mRNA) is the putative DSE of the A2 site. Although alleles of this site (T_22-27_ and T_12_GT _3_GT_6_) are in linkage disequilibrium (LD) with rs12079745 (D’ = 0.73), this TRS is independently associated with BP in a European-American population. By *in vitro* assays, we demonstrate that this allele mediates its effect via increasing polyadenylation at the A2 site. Furthermore, we show higher relative abundance of the A2-polyadenylated mRNA in human kidneys heterozygous for the T_12_GT _3_GT_6_ allele than in T_23_/T_23_ kidneys. Therefore, we have identified a common human polymorphism in the DSE of *ATP1B1* that may contribute to BP regulation by regulating *ATP1B1* mRNA processing.

## Materials and Methods

### Subjects

Samples used in the association study were ascertained through the GenNet network of the Family Blood Pressure Program [9]. Phenotype characteristics of the GenNet samples have been previously published [9]. Briefly, subjects’ ages ranged between 18–50 years. Their mean SBP and DBP were 123.2 ± 17.6 and 77.0 ± 10.0 mmHg, respectively. Overall, 32% were clinically hypertensive with SBP/DBP over 140/90 mmHg or were taking anti-hypertension medication. To measure *ATP1B1* expression, we used RNA from Epstein Barr Virus (EBV)-transformed lymphocytes from healthy Amish subjects from the Amish Family Longevity Study (AFLS) [28], and human kidneys from the National Disease Research Interchange, Philadelphia, PA (http://ndriresource.org/). Approval for the collection and use of the AFLS samples was provided by the University of Maryland, Baltimore (UMB) Institutional Review Board (IRB). Written informed consent was obtained from AFLS subjects for the use of their samples. The kidney samples used were exempted from IRB approval by the UMB IRB.

### Genotyping

The TRS was amplified from human genomic DNA using the following primers: F-5’-Gacaaaagaaaaagaaaaattgagc-3’ and R-5’-aaataaagacctaacaccacaggaa-3’. Sequencing was performed using the BigDye Terminator reaction chemistry (Applied Biosystems, Foster City, CA) according to the manufacturer’s recommendations. Sequences were analyzed using Sequencher 4.5 (Gene Codes, Ann Arbor, MI). The length of the TRS was deduced by electrophoresis of fluorescently labelled PCR product along with size markers. Genomic DNA was amplified using the following primers: F-5’-CTGTTTTCTACTTTATGTGAGCAAGG-3’ (FAM labelled) and R-5’-CTCATCGATGGGCCATTAC-3’ and the GoTaq Colourless Master Mix (Promega, Madison, WI). Size was determined by electrophoresis of the PCR products mixed with Hi-Di Formamide (Applied Biosystems) and the Gene Scan 600Liz size standard (Applied Biosystems). The results were analyzed using the GeneMapper 4.0 software (Applied Biosystems) and examples of the electropherograms of T_23_ and T_24_ are shown in Figure S1.

### Polyadenylation assay

**Figure 2:**
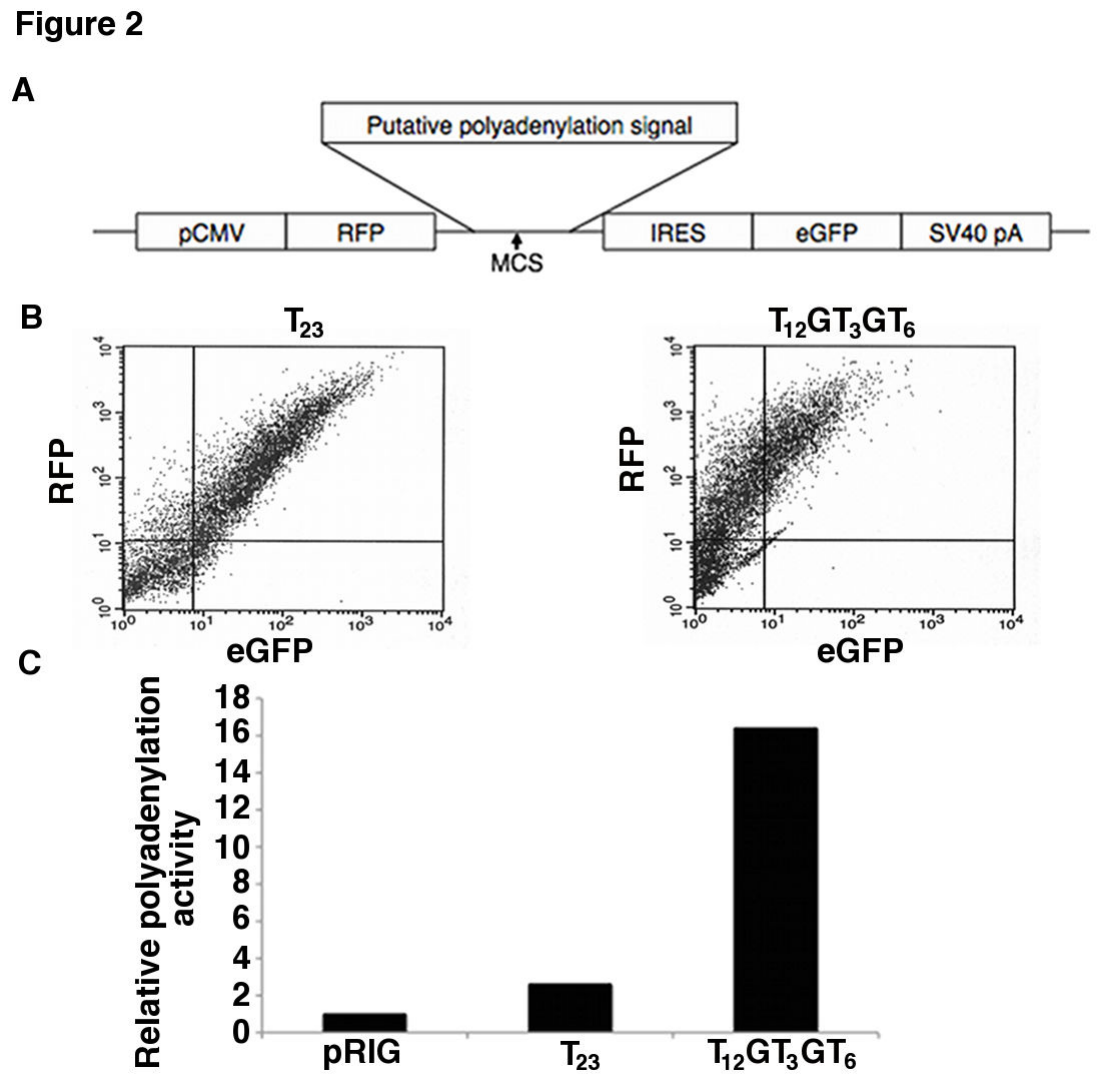
The T_12_GT _3_GT_6_ allele increases polyadenylation at the A2 polyadenylation site. A) Schematic of the pRIG vector. MCS: multiple cloning site, where the tested sequences were inserted, pCMV: cytomegalovirus minimal promoter, RFP: red fluorescent protein, IRES: internal ribosomal entry site, eGFP: enhanced green fluorescent protein, SV40 pA: SV40 polyadenylation signal. B) Dot plots from FACS analysis of the expression of RFP and eGFP in HEK293T cells transfected with the T_23_ and T _12_GT _3_GT_6_ alleles in the pRIG vector. Each dot represents a cell and the X- and Y-axes are the fluorescence intensity of eGFP and RFP, respectively. C) The ratio of RFP to eGFP expression in pRIG with no insert, T_23_, and T_12_GT _3_GT_6_.

A 372 base pair (bp) fragment of the *ATP1B1* 3’UTR spanning the TRS was amplified from genomic DNA using the following primers: F-5’-GACAAAAGAAAAAGAAAAATTGAGC-3’, R-5’-AAATAAAGACCTAACACCACAGGAA-3’. The products were cloned into the pCR4-TOPO vector (Invitrogen) and then into the pRIG vector (Figure 2A) using *EcoR*I digestion and ligation. Successful cloning was verified by colony PCR and sequencing. Polyadenylation activity was determined as described in Pan et al. [30]. Briefly, Human Embryonic Kidney 293T (HEK293T) cells grown in 6-well plates were transfected with the various pRIG vectors (1 µg plasmid in each well) using the Fugene 6 transfection kit (Roche). Cells were harvested 48 h after transfection by treatment with Trypsin-EDTA. When cells are transfected with the empty pRIG vector, red and green fluorescence proteins (RFP and eGFP, respectively) are equally expressed. Insertion of a polyadenylation signal-containing sequence into pRIG at the multiple cloning site (MCS) leads to the cleavage of the transcript between the RFP and eGFP open reading frames (ORFs) causing cells to express more RFP than eGFP. The ratio of RFP to eGFP in cells reflects the strength of the polyadenylation signal tested. The relative abundance of RFP and eGFP were measured at 530 nm and 585 nm, respectively, using the FACScalibur system (BD Biosciences, San Jose, CA).

### RNA isolation and real-time PCR

**Figure 3:**
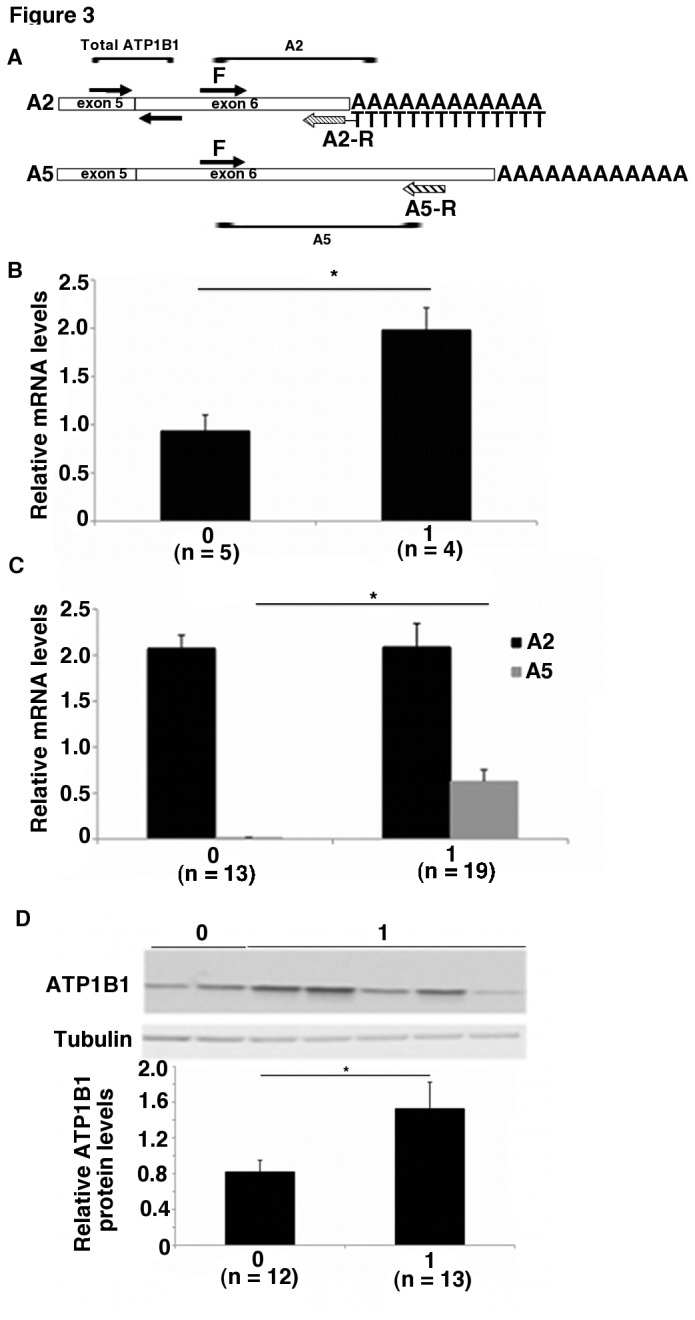
The T_12_GT _3_GT_6_ allele regulates the polyadenylation of *ATP1B1* mRNA in human tissues A) Schematic of the primer design used to distinguish between the A2- and A5- polyadenylated mRNAs. The A2-R primer had a 5’ T_15_ tail to selectively amplify A2-polyadenylated mRNA. Total *ATP1B1* mRNA was measured using primers that amplified across the exon 5/exon 6 junction. B) Real-time PCR of mRNA from human kidneys. The A2-polyadenylated transcript was quantified relative to total *ATP1B1* mRNA. 0 and 1 indicate subjects without the T _12_GT _3_GT_6_ allele and with 1 copy of the T _12_GT _3_GT_6_ allele, respectively. Error bars represent standard errors. *p < 0.0001. C) Real-time PCR of mRNA from human lymphocytes. Expression of the A2- and A5-polyadenylated mRNAs relative to total *ATP1B1* mRNA in lymphocytes from subjects with 0 or at least 1 copy of the T _12_GT _3_GT_6_ allele. *p = 0.0001 between the A5-polyadenylated transcript levels in the 2 groups. Data represent the average across samples from each genotype group with standard error. D) Expression of ATP1B1 protein in lymphocytes from individuals with 0 or at least 1 copy of the T _12_GT _3_GT_6_ allele. Tubulin was used as a loading control. The graph quantifies the average level of ATP1B1 protein relative to tubulin with standard error. *p = 0.05.

RNA was isolated from sections of frozen kidneys and EBV-transformed lymphocytes using the RNeasy Mini Kit from Qiagen (Valencia, CA) according to the manufacturer’s instructions. RNA was treated with DNAse I, Amplification grade (Life Technologies, Grand Island, NY) and reverse-transcribed using oligodT primers and the Superscript III First-Strand Synthesis Supermix (Life Technologies). Real-time PCR was performed using the LightCycler 480 SYBR Green I Master (Roche, Indianapolis, IN) on a Roche LightCycler 480. The following primers were used to amplify the A2- and A5-polyadenylated mRNAs and are indicated in Figure 3A: A2/A5-F-5’-CTGGCCCCTAAGTATTGCT-3’, A2-R-5’-TTTTTTTTTTTTTTACCATGCCAGTTTTATTC-3’, A5-R-5’-CACCACAGGAAAAGACTATGGA-3’. The reverse primer for the A2-polyadenylated mRNA had an additional 5’ T_14_ adaptor in order to selectively amplify A2-polyadenylated mRNA. Melt curve analysis was used to ensure a specific product. Expression was normalized to total *ATP1B1* transcript levels (amplified using primers 5’-AATGTCCTTCCCGTTCAGTG-3’ and 5’-CAGAGGAAAACCAGGGGAGT-3’) due to the known differences in *Atp1b1* mRNA levels in different regions of the kidney [29]. All transcripts were quantified based on standard curves generated from pooled cDNA and using the LightCycler 480 Relative Quantification software (Roche). Each sample was tested in triplicate and in at least 2 independent experiments. Data from subjects without the T_12_GT _3_GT_6_ allele (0) and those with at least 1 copy of the T_12_GT _3_GT_6_ allele (1) are presented in all figures. Significance was calculated using Student’s *t*-test. *p* < 0.05 was considered significant.

### Genetic analysis

Association analyses were performed using MMAP (http://edn.som.umaryland.edu/mmap/index.php) [31]. Briefly, this software uses a variance component model that assesses the effect of genotype, as an additive effect, on the phenotype of interest while simultaneously estimating the effects of age, age^2^, sex, and a polygenic component to account for phenotypic correlation due to relatedness. An (n-1)-degree-of-freedom *t* test was used to assess the significance of the measured genotype. The genotype was coded as the dosage of the effect alleles shown in Table 1, with the remaining alleles being collapsed to the non-effect allele. For those GenNet subjects taking anti-hypertension medication (22%), analyses were performed either by removing these individuals or by adding the average treatment effect to raw BP readings (10 and 5 mmHg to SBP and DBP, respectively) [32]. LD calculations between TRS and rs12079745 were made using 2LD [33,34]. Sequence alignment was performed using ClustalW [35,36] and orthologous sequences were obtained from the UCSC genome browser (www.genome.ucsc.edu) from assemblies hg18, panTro2, rheMac2, mm9, rn4, bosTau4, canFam2, monDom5, oviAri1, and myoLuc2. The pteVam1 sequence was obtained from scaffold_1928 from Ensembl (www.ensembl.org).

**Table 1:** Association analysis of the TRS alleles with blood pressure.

			Adjusted for medication				Unmedicated only			
Model	Variant	Effect allele	SBP (n = 830)		DBP (n = 831)		SBP (n = 647)		DBP (n = 648)	
			Effect size*	*p* value	Effect size	*p* value	Effect size	*p* value	Effect size	*p* value
Single locus	rs12079745	A	2.8	0.10	1.0	0.32	4.9	0.001	2.4	0.022
	TRS	T_12_GT_3_GT_6_	3.3	0.014	2.4	0.003	2.1	0.10	1.9	0.029
Joint Analysis	rs12079745	A	3.2	0.07	1.3	0.21	5.1	0.001	2.6	0.014
	TRS	T_12_GT_3_GT_6_	3.6	0.009	2.5	0.002	2.4	0.06	2.1	0.019

SBP: systolic blood pressure, DBP: diastolic blood pressure, *effect sizes are in mmHg

### Protein extraction and Western blot analysis

Protein was extracted by sonicating lymphocytes in lysis buffer (25 mM Tris-HCl, pH 7.5, 1% SDS, protease inhibitor tablet [Quality Biologicals, Gaithersburg, MD], and PIC). Protein was quantified using the BCA Protein Assay Kit (Thermo Scientific, Rockford, IL) according to the manufacturer’s instructions. Twenty micrograms of protein was deglycosylated using PNGaseF (New England Biolabs, Ipswich, MA). The deglycosylated lysates were separated on a 10% polyacrylamide gel (Bio-Rad, Hercules, CA) and transferred to a polyvinylidene difluoride membrane (PVDF, Bio-Rad). The membrane was blocked overnight, treated with a monoclonal ATP1B1 antibody (1:5000, MA3-930, Thermo Scientific) for 1 h at room temperature, washed and incubated with an anti-mouse secondary antibody (1:5000, 115-035-003, Jackson ImmunoResearch) for 1 h at room temperature. The membrane was developed using the Chemiluminescent Western blotting kit (Thermo Scientific) and the protein was visualized on the FluorChem Q system (ProteinSimple, Santa Clara, CA). When needed, the membrane was stripped with Restore Plus Western Blot Stripper Buffer (Thermo Scientific). ATP1B1 protein was quantified relative to tubulin (1:2000, T6199, Sigma-Aldrich) using the AlphaView-FluorChem Q version 3.2.2 software (ProteinSimple). The experiment was repeated twice and yielded consistent results. Once normalized, data from all experiments are presented as mean across samples from the same genotype group with standard error. Data from subjects without the T_12_GT _3_GT_6_ allele (0) and those with at least 1 copy of the T_12_GT _3_GT_6_ allele (1) are presented in all figures. Significance was calculated using Student’s *t*-test. A *p* value < 0.05 was considered significant.

## Results

### A multi-allelic T-rich sequence in the 3’UTR of ATP1B1 is a putative DSE

Examination of the 3’UTR of *ATP1B1* revealed a highly polymorphic T-rich sequence (TRS) 13 nucleotides downstream of the A2 polyadenylation signal (Figure 1A). This TRS has 22 T nucleotides in the reference genome (hg18), but the presence of 5 annotated biallelic SNPs and insertions/deletions in dbSNP (rs34447553, rs1779795, rs1779796, rs56167655, and rs4656649) suggests that this TRS might be structurally complex and contain multiple alleles. To identify sequence variants in the TRS, we sequenced this region in 1,010 European American samples collected through the GenNet network. We identified 7 alleles in this TRS that vary the length of the T-track and the number of G nucleotides that interrupt the T-track: T_22_, T_23_, T_24_, T_25_, T_26_, T_27_, and T_12_GT _3_GT_6_ (representative sequence traces of T_23_ and T_12_GT _3_GT_6_ are shown in Figure 1B). The most common alleles were T_23_ and T_12_GT _3_GT_6_ with allele frequencies of 0.82 and 0.12, respectively. The remaining alleles had allele frequencies ranging from 0.04 to 0.001.

Sequence inspection suggested a potential functional role for the TRS alleles. Species in which the A2 polyadenylation signal adheres to the AATAAA consensus polyadenylation sequence have a T-rich sequence downstream of the polyadenylation signal, whereas species such as microbat that have a variant polyadenylation signal have a relatively T-poor region downstream of the polyadenylation site (Figure 1C) [37]. This suggests that the TRS and the A2 polyadenylation signal may be functionally linked, and that the TRS may encode the uridine-rich element that serves as the DSE of the A2 polyadenylation site. In fact, this TRS contains a putative CstF binding site that is 72.2% similar to the 2a consensus CstF binding site (Figure 1D) [38]. Interestingly, the T_12_GT _3_GT_6_ sequence is more similar to the 2a consensus CstF binding site than the alleles with pure T-track (83.3% vs 72.2%, Figure 1D). Therefore, we investigated whether the TRS alleles may be responsible for the association signal seen in *ATP1B1* in our previous study, and whether any such association may be mediated through the effect of these alleles on the polyadenylation of the *ATP1B1* mRNA.

### Alleles of the TRS are associated with BP independently of rs12079745

In order to determine whether the TRS alleles have an effect on BP, we first performed an association analysis of the TRS alleles with systolic BP (SBP) and diastolic BP (DBP) in the GenNet samples in which the original association was identified. Subjects on HTN medication either had their SBP and DBP adjusted (see Methods) or were excluded from the analysis. Of primary interest were the T_23_ and T_12_GT _3_GT_6_ alleles because they were the most common alleles in this population. Since too few subjects carried alleles T_22_, T_24_, T_25_, T_26_, and T_27_ to provide sufficient power for association analysis, we analyzed our data by collapsing all non-T _12_GT _3_GT_6_ alleles as the reference allele (Table 1) and by removing subjects carrying alleles other than T_23_ and T_12_GT _3_GT_6_ from the analysis (Table S1).

As shown in Table 1, when analyzed individually, rs12079745 and the TRS were both significantly associated with SBP and DBP (Single locus model). The rs12079745 A allele was associated with increased SBP (beta = 4.9, *p* = 0.001) and increased DBP (beta = 2.4, *p* = 0.022) in unmedicated subjects, but not in the full sample. On the other hand, the TRS T_12_GT _3_GT_6_ allele was associated with SBP (beta = 3.3, *p* = 0.014) and DBP (beta = 2.4, *p* value = 0.003) in the full sample, and only DBP (beta = 0.19, *p* = 0.029) in unmedicated subjects. Removing alleles with low allele frequencies gave a similar result (Table S1). Thus, both rs1207974 and TRS were significantly associated with DBP in unmedicated subjects. To better understand if the association of both loci with DBP were independent we performed multiallelic LD and joint association analyses. These 2 loci were in LD (D’ = 0.73, SD = 0.04) and the most common haplotypes were G-T_23_ and G-T _12_GT _3_GT_6_, with haplotype frequencies of 0.80 and 0.11, respectively. All other remaining haplotypes had estimated frequencies <0.03, and the haplotype A-T _12_GT _3_GT_6_ containing both higher BP-associated alleles was not observed. We then compared the joint model for unmedicated DBP containing both rs1209745 and TRS with each single locus model to determine if the *p* value of the second locus remained significant or was attenuated when included in the model. Table 1 shows that the *p* values become smaller and the effect sizes become larger in the joint model, thus showing the loci are independent. In fact, the joint model for the other 3 phenotypes provides a slightly better fit than either single locus model, suggesting the rs12079745 and TRS may have independent effects on BP.

### T_12_GT_3_GT_6_ causes increased polyadenylation at the A2 polyadenylation site as compared to T_23_

To determine whether the more common T_12_GT _3_GT_6_ and T_23_ alleles had specific effects on polyadenylation at the A2 polyadenylation site, we tested the polyadenylation activity of these alleles in a vector (pRIG) designed specifically to test polyadenylation efficiency [30]. The structure of the pRIG vector is shown in Figure 2A. As shown in Figure 2B and 2C, the polyadenylation activity of the T_23_ allele was approximately twice that of the empty pRIG vector, validating the A2 polyadenylation signal with the TRS as a *bona fide* polyadenylation site. Furthermore, more cells expressed a higher ratio of RFP:eGFP in the presence of the T_12_GT _3_GT_6_ allele than that of the T_23_ allele, indicating that more cleavage and polyadenylation occurred in the presence of the T_12_GT _3_GT_6_ allele. Quantification of this activity showed that the polyadenylation activity of the T_12_GT _3_GT_6_ allele was approximately 6-fold greater than that of the T_23_ allele in HEK293T cells. The presence of the A versus G allele of rs12079745 did not alter polyadenylation activity (data not shown). Therefore, these results show that the T_12_GT _3_GT_6_ allele at the TRS increases polyadenylation at the A2 polyadenylation site as compared to the T_23_ allele.

### Human tissues with and without the T_12_GT_3_GT_6_ allele exhibit differential polyadenylation of ATP1B1 mRNA

Next, we tested whether the T_12_GT _3_GT_6_ allele also regulates alternative polyadenylation of *ATP1B1 in vivo*. We determined the abundance of the A2- and A5-polyadenylated *ATP1B1* mRNAs relative to total *ATP1B1* levels in human kidneys (see Methods). Human kidneys express high levels of ATP1B1 [21], and are intimately involved in BP regulation [39]. Among the human kidneys available, 4 were heterozygous for the T_12_GT _3_GT_6_ (T_12_GT _3_GT _6_/T_23_) allele while the rest were homozygous T_23_/T_23_. All the kidneys used were homozygous for the G allele at rs12079745. We measured the relative abundance of the A2- and A5-polyadenylated mRNAs in these 4 heterozygotic kidneys and compared it to that of 5 T _23_/T_23_ kidneys by quantitative PCR using the primers depicted in Figure 3A. As shown in Figure 3B, the relative abundance of the A2-polyadenylated mRNA was approximately twice as high in kidneys with the T_12_GT _3_GT_6_ allele than in those lacking T_12_GT _3_GT_6_ (*p* < 0.0001). The A5-polyadenylated transcript was undetectable in the kidneys tested.

We also quantified the relative abundance of the A2- and A5-polyadenylated mRNAs in lymphocytes, for which more samples overall and more carriers of the T_12_GT _3_GT_6_ allele were available. Lymphocytes also express Na-K ATPase pumps, albeit at a lower level than skeletal muscle and kidney tissue [15]. Furthermore, *ATP1B1* mRNA levels have been shown to be detectable at higher levels in mature lymphocytes than in immature lymphocytes [40]. Since only 3 samples were homozygous for the T_12_GT _3_GT_6_ allele, we combined data from the heterozygote and homozygote T_12_GT _3_GT_6_ samples for the subsequent analysis. In contrast to human kidneys, in lymphocytes, the relative abundance of the A2-polyadenylated mRNA was independent of TRS genotypes, whereas the abundance of the A5-polyadenylated mRNA was significantly higher in the presence of the T_12_GT _3_GT_6_ allele (*p* = 0.0001) (Figure 3C). In lymphocytes, this increase in the relative abundance of the A5-polyadenylated mRNA translated into a significant increase in ATP1B1 protein levels in the presence of the T_12_GT _3_GT_6_ allele (*p* = 0.05) (Figure 3D). Together, these results suggest that the TRS alleles modulate alternative polyadenylation of *ATP1B1* transcripts in human tissues but their effects on mRNA isoform abundance differ across tissue types.

## Discussion

We have identified a novel polymorphic and functional TRS in the 3’UTR of *ATP1B1*. Using human tissue samples and *in vitro* assays, we have shown that the alleles of this TRS regulate the alternative polyadenylation of *ATP1B1*. We also showed that the alleles are associated with BP in a European-American population. Therefore, the TRS alleles likely regulate BP by regulating alternative polyadenylation of *ATP1B1*.

Polymorphisms in the TRS likely mediate their effect on polyadenylation by modulating the binding of cleavage stimulating factors. The T_12_GT _3_GT_6_ allele of the TRS is more similar to the consensus binding site of the cleavage stimulating factor CstF64 than the T_23_ allele (Figure 1D) [38]. While Takagaki and Manley found that both GU- and U-rich sequences are recognized by CstF64 [41], the yeast homolog of CstF64, Rna15, was found to have a preference for GU-containing RNA [42], suggesting that the GUs in T_12_GT _3_GT_6_ may increase the affinity of the TRS for CstF64, thereby enhancing A2 polyadenylation site usage. Such an increased affinity for CstF64 may in turn lead to the increased abundance of the A2-polyadenylated *ATP1B1* mRNA. In fact, regulation of CstF64 binding has been shown to regulate the switch between the synthesis of the secreted and membrane bound forms of immunoglobulin [43]. Interestingly, while multiple alleles that vary the length of the T-track were found, we did not find any alleles similar to T_12_GT _3_GT_6_ that varied the length of the T-track, such as T_14_GT _3_GT_6_, again suggesting that the spacing of the 2 additional G nucleotides is functionally important.

Alternatively, sequence variants in the TRS may also cause changes to the secondary structure of *ATP1B1* mRNA. The RNA folding program MFOLD predicts differences in the secondary structure of *ATP1B1* mRNA containing the U_23_ and U_12_GU _3_GU_6_ sequences (Figure S2 and Information S1) [44]. Effects on mRNA 3’ end processing or mRNA stability, mediated through changes in the secondary structure of either the A5-polyadenylated mRNA or the premature mRNA, may contribute to the different levels of A5-polyadenylated mRNA noted in lymphocytes. Further studies will be required to determine if the T_12_GT _3_GT_6_ allele impacts *ATP1B1* mRNA post-transcriptionally through mechanisms other than polyadenylation.

Our association analyses showed that while rs12079745 and the TRS are in LD, these 2 sites are independently associated with BP. The effect or higher BP-associated allele of the TRS (T_12_GT _3_GT_6_) and that of the SNP (A) are not found on the same haplotype in our study population. Furthermore, the association between rs12079745 and SBP is stronger than that between this SNP and DBP, whereas the TRS is more strongly associated with DBP than with SBP. In fact, having both loci in the same model provides stronger association to BP than having only one locus in the model. While rs12079745 is in strong LD with several other SNPs in and near the 3’UTR of *ATP1B1*, none of these SNPs are located in a sequence context in which a potential function can be computationally predicted and experimentally tested. However, these SNPs might contribute to the regulation of *ATP1B1* expression and BP levels via other 3’UTR functions unrelated to alternative polyadenylation. Indeed, Wei et al. demonstrated the role for a common 3’UTR variant in *ATP6V0A1* that alters hypertension risk by creating a microRNA binding site that affects *ATP6V0A1* mRNA expression [45]. Additional studies are needed to determine how rs12079745 or another site in LD with this SNP may regulate *ATP1B1* expression and explain the association between rs12079745 and BP.

Since the β1 subunit encoded by *ATP1B1* is the rate-limiting factor in the assembly and activity of Na-K ATPase, an increase in *ATP1B1* mRNA levels in the presence of the T_12_GT _3_GT_6_ allele is expected to increase the abundance and function of Na-K ATPase activity [46]. Increased Na-K ATPase activities have been correlated with higher BP through studies in animal models. In the Milan Hypertensive Rat, an increase in *ATP1B1* expression and Na-K ATPase activity in the kidney precedes the onset of hypertension [47,48]. Knock-in gene targeting of the alpha-2 isoform of Na-K ATPase in mice makes them insensitive to ouabain-induced hypertension [49]. A conditional cardiac knockout of *Atp1b1* in a mouse model also demonstrated that *Atp1b1* is crucial for cardiac growth, contractility, and ouabain-sensitivity [50]. Furthermore, a 2-kidney, 1-clip rat model of hypertension also showed increased Na-K ATPase levels and increased phosphorylation of Na-K ATPase in renovascular hypertension [51]. The importance of Na-K ATPase in human essential hypertension, due to its heterogenous condition, is less well established. However, rostafuroxin, a compound that lowers blood pressure by restoring normal renal and vascular Na-K ATPase functions, is currently in clinical trial and might be effective in a genotype-specific manner [52]. Measuring the effect of the TRS alleles on Na-K ATPase activity was not feasible because the TRS appears to influence the polyadenylation of *ATP1B1* transcripts in a tissue specific manner and human tissues most relevant for blood pressure homeostasis were not available for study. Therefore, further studies will be required to determine the effect of the identified variants on Na-K ATPase activity.

While the A5-polyadenylated mRNA is less abundant than the A2-polyadenylated mRNA in most human tissues that express high levels of *ATP1B1* (Figure S3 and Information S1), we were unable to detect A5-polyadenyated mRNA in our collection of kidneys. This can be due to the low abundance of this mRNA in the kidney or suboptimal quality of RNA obtained from the kidneys used in this study. The frozen kidneys from which RNA was extracted in this study were obtained post-mortem, from donors who died from a variety of causes unrelated to acute kidney diseases. Although the overall quality of the RNAs was acceptable for standard molecular analysis, the intactness of transcripts with destabilizing motifs, such as those present in the A5-polyadenylated mRNA, is unknown. Furthermore, the kidney is a complex organ and many genes are differentially regulated throughout the nephron [29]. The kidney sections available to us varied in composition and tissue heterogeneity (versus studying only renal cortex, for example). This may have contributed to our inability to detect the A5-polyadenylated transcript. Therefore, we cannot exclude the possibility that technical limitations prevented us from determining how the change in the relative abundance of the A2-polyadenylated mRNA affects that of the A5-polyadenylated mRNA in human kidneys. Nevertheless, we were still able to detect an increase in A2-polyadenylated mRNA levels in kidneys containing the T_12_GT _3_GT_6_ allele, suggesting that the T_12_GT _3_GT_6_ allele does indeed have an effect on *ATP1B1* polyadenylation.

In contrast, the A5-polyadenylated mRNA was detectable in freshly harvested lymphocytes, where only the relative abundance of the A5-polyadenylated mRNA was higher in the presence of the T_12_GT _3_GT_6_ allele. This may be due to the preferential use of one polyadenylation site versus another in different tissues. Indeed, over 70% of tandem 3’UTR polyadenylation sites in humans have been shown to be used in a tissue-specific manner [53]. Furthermore, the lower abundance of Na-K ATPase pumps in lymphocytes versus in kidneys may contribute to differences in the regulation of the *ATP1B1* gene in lymphocytes versus kidneys [15].

Our study is one of the few examples, to our knowledge, of a polymorphic DSE involved in allele-specific gene expression via alternative polyadenylation that is associated with a clinically important phenotype. Uitte de Willige et al., found a C>T transversion (TACCT>TATCT, rs2066865) in the DSE of the fibrinogen γ (*FGG*) gene that has an impact on the putative CstF64 binding site of *FGG* similar to what we see with the T_23_ to T_12_GT _3_GT_6_ change in the TRS [54]. This TATCT allele is also relatively common (0.22 and 0.50 in HapMap European-Americans and Asians, respectively), alters the fibrinogen γ’/γA ratio by regulating the alternative polyadenylation of *FGG* mRNA, and is associated with an increased risk of deep vein thrombosis [54,55]. Another example is provided by a rare gain-of-function C20221T mutation 11 bp downstream of the cleavage site of the prothrombin *F2* gene. This mutation alters a TGCT sequence to a TGTT motif and shows greater mRNA expression than the wild-type sequence and was found in patients with abnormal thrombosis [56]. Given that an estimated 5–10% of all GWA hits are located in the 3’UTR [57], and 50% of all protein-coding genes possess multiple polyadenylation signals and are partially regulated through alternative polyadenylation [58], there are certainly more such variants to be uncovered that are clinically relevant and/or can yield important biological insights. In fact, many risk alleles from GWAS studies are correlated with alleles that can cause alternate polyadenylation [59]. Because genotyping this complex TRS site is more technically demanding than SNP genotyping and sequencing, the allele frequency of the TRS in other populations is still unknown. In fact, we identified another allele, T_16_GT_6_, in African Americans (data not shown) but its impact on polyadenylation and BP is still unknown. Furthermore, simple repeat sequences, such as the TRS in ATP1B1, are often prone to mutate. Given how polymorphic this TRS is (8 alleles identified so far), it is highly likely that other DSEs are also polymorphic and that the regulation of alternative polyadenylation may be a relatively common mechanism in the susceptibility of quantitative traits, such as BP, across human populations.

We have identified novel polymorphisms in the 3’UTR of *ATP1B1* that are associated with BP. We have also shown that these polymorphisms alter what is most likely the DSE of the gene, and that these polymorphisms mediate changes in the relative abundance of differentially polyadenylated *ATP1B1* transcripts by regulating alternative polyadenylation of the gene.

## Supporting Information

Figure S1 **Determination of the T track genotypes.** Electropherograms from GeneMapper for the T_23_ and T_24_ alleles.
(TIF)

Figure S2 **Predicted mRNA secondary structures of the *ATP1B1* mRNA transcripts with the U_23_ and U_12_GU _3_GU_6_ sequences.** (A) MFOLD prediction of the secondary structure of the mRNA transcript containing the U_23_ sequence. (B) MFOLD prediction of the secondary structure of the mRNA transcript containing the U_12_GU _3_GU_6_ sequence. The locations of A2 polyadenylation signal sequence and the TRS are indicated.
(TIF)

Figure S3 **Expression of the A2- and A5-polyadenylated *ATP1B1* transcripts in a human tissue panel.** Real-time PCR analysis of the levels of the A2- and A5-polyadenylated *ATP1B1* transcripts relative to GAPDH mRNA levels in a human tissue panel.
(TIF)

Information S1 **Supporting Methods.**
(DOC)

Table S1 **Association analysis of only the T_23_ and T_12_GT _3_GT_6_ alleles with blood pressure.** Other alleles with frequencies <0.05 were removed from the model.
(DOC)
