# Supplementary figures and images for: A Polymorphic 3’UTR Element in ATP1B1 Regulates Alternative Polyadenylation and Is Associated with Blood Pressure
Source: PLoS One. 2013 Oct 1;8(10):e76290. doi: 10.1371/journal.pone.0076290 (PMC3788127; doi:10.1371/journal.pone.0076290)

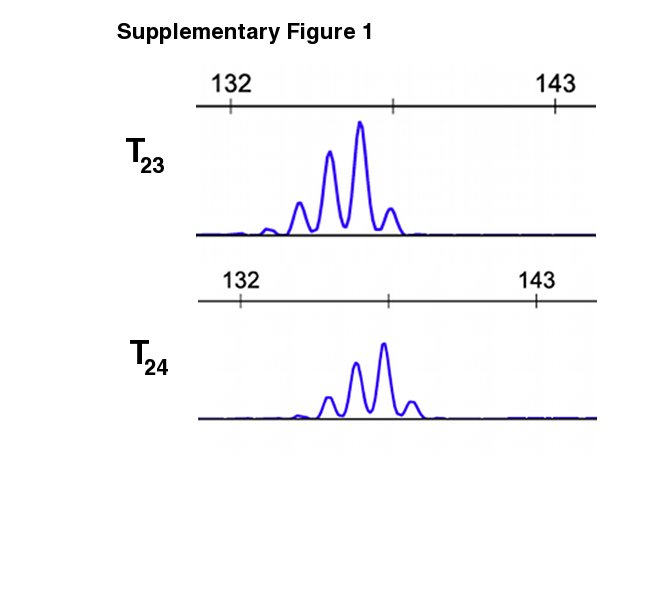

Supplement: Figure S1 — Determination of the T track genotypes. Electropherograms from GeneMapper for the T23 and T24 alleles. (TIF) [file pone.0076290.s001.tif]

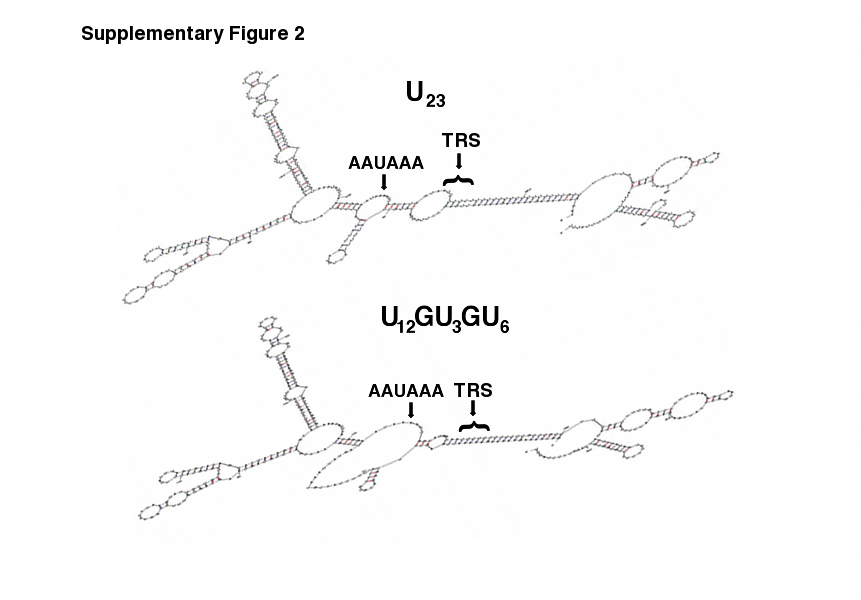

Supplement: Figure S2 — Predicted mRNA secondary structures of the ATP1B1 mRNA transcripts with the U23 and U12GU 3GU6 sequences. (A) MFOLD prediction of the secondary structure of the mRNA transcript containing the U23 sequence. (B) MFOLD prediction of the secondary structure of the mRNA transcript containing the U12GU 3GU6 sequence. The locations of A2 polyadenylation signal sequence and the TRS are indicated. (TIF) [file pone.0076290.s002.tif]

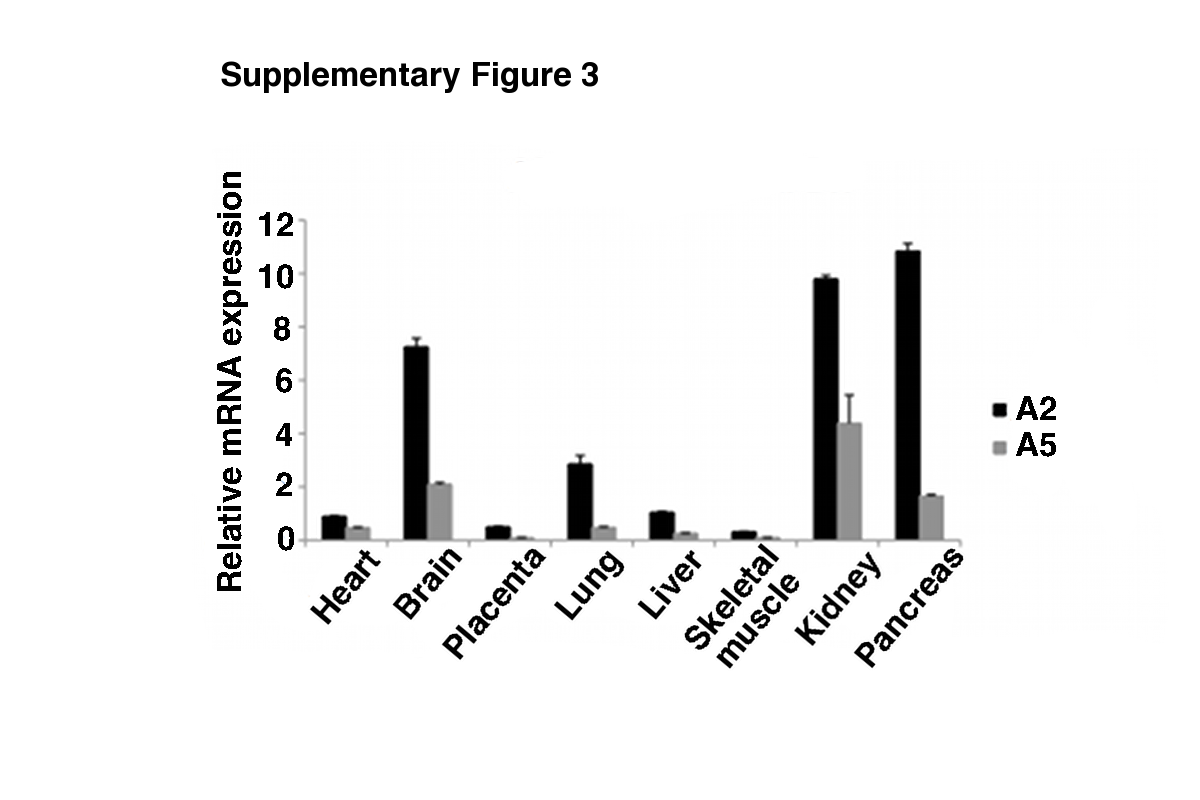

Supplement: Figure S3 — Expression of the A2- and A5-polyadenylated ATP1B1 transcripts in a human tissue panel. Real-time PCR analysis of the levels of the A2- and A5-polyadenylated ATP1B1 transcripts relative to GAPDH mRNA levels in a human tissue panel. (TIF) [file pone.0076290.s003.tif]
