# Supplementary material for: A Polymorphic 3’UTR Element in ATP1B1 Regulates Alternative Polyadenylation and Is Associated with Blood Pressure
Source: PLoS One. 2013 Oct 1;8(10):e76290. doi: 10.1371/journal.pone.0076290 (PMC3788127; doi:10.1371/journal.pone.0076290)
Supplement: Information S1 — Supporting Methods. (DOC) [file pone.0076290.s004.doc]

**SUPPORTING INFORMATION**

**METHODS**

**RNA Folding**

RNA folding was predicted using MFOLD and the sequence of the mature A5-polyadenylated *ATP1B1* mRNA transcript and default parameters. ^1^

**Real-time PCR analysis in the human tissue panel**

A multiple human tissue cDNA panel (Clontech, Mountain View, CA) was used to quantify the A2-and A5-polyadenylated *ATP1B1* transcript levels using the following primers: A2/A5-F-5’-ctggcccctaagtattgct-3’, A2-R-5’-ttttttttttttttaccatgccagttttattc-3’, A5-R-5’-caccacaggaaaagactatgga-3’. Transcript levels were normalized to GAPDH, which was amplified using the following primers: GAPDH-F-5’-tgatgacatcaagaaggtggtgaag-3’ and GAPDH-R-5’- tccttggaggccatgtgggccat-3’. All transcripts were quantified based on standard curves generated from pooled cDNA and using the LightCycler 480 Relative Quantification software (Roche, Indianapolis, IN). Each sample was tested in triplicate and in at least 2 independent experiments.

**REFERENCES**

1. Zuker, M., Mfold web server for nucleic acid folding and hybridization prediction, Nucleic Acids R**es** 31, 3406-15 (2003).
