# Supplementary material for: A Polymorphic 3’UTR Element in ATP1B1 Regulates Alternative Polyadenylation and Is Associated with Blood Pressure
Source: PLoS One. 2013 Oct 1;8(10):e76290. doi: 10.1371/journal.pone.0076290 (PMC3788127; doi:10.1371/journal.pone.0076290)
Supplement: Table S1 — Association analysis of only the T23 and T12GT 3GT6 alleles with blood pressure. Other alleles with frequencies <0.05 were removed from the model. (DOC) [file pone.0076290.s005.doc]

|  | | | Adjusted for medication | | | | Unmedicated only | | | |
| --- | --- | --- | --- | --- | --- | --- | --- | --- | --- | --- |
| Model | Variant | Effect allele | SBP (n = 722) | | DBP (n = 723) | | SBP (n = 562) | | DBP (n = 563) | |
|  |  |  | Effect size* | *p* value | Effect size | *p* value | Effect size | *p* value | Effect size | *p* value |
| Single Locus Analysis | TRS | T_12_GT_3_GT_6_ | 3.8 | 0.006 | 2.6 | 0.002 | 2.5 | 0.06 | 2.3 | 0.012 |

**Table S1. Association analysis of only the T_23_ and T_12_GT_3_GT_6_ alleles with blood pressure. Other alleles with frequencies <0.05 were removed from the model.**

SBP: systolic blood pressure, DBP: diastolic blood pressure, *effect sizes are in mmHg
